# Supplementary material for: Multi-Omics Analysis in Mouse Primary Cortical Neurons Reveals Complex Positive and Negative Biological Interactions Between Constituent Compounds of Centella asiatica
Source: Pharmaceuticals (Basel). 2024 Dec 27;18(1):19. doi: 10.3390/ph18010019 (PMC11768890; doi:10.3390/ph18010019)
Supplement: Supplementary file 1 [file pharmaceuticals-18-00019-s001.zip › Figure S1.pdf]

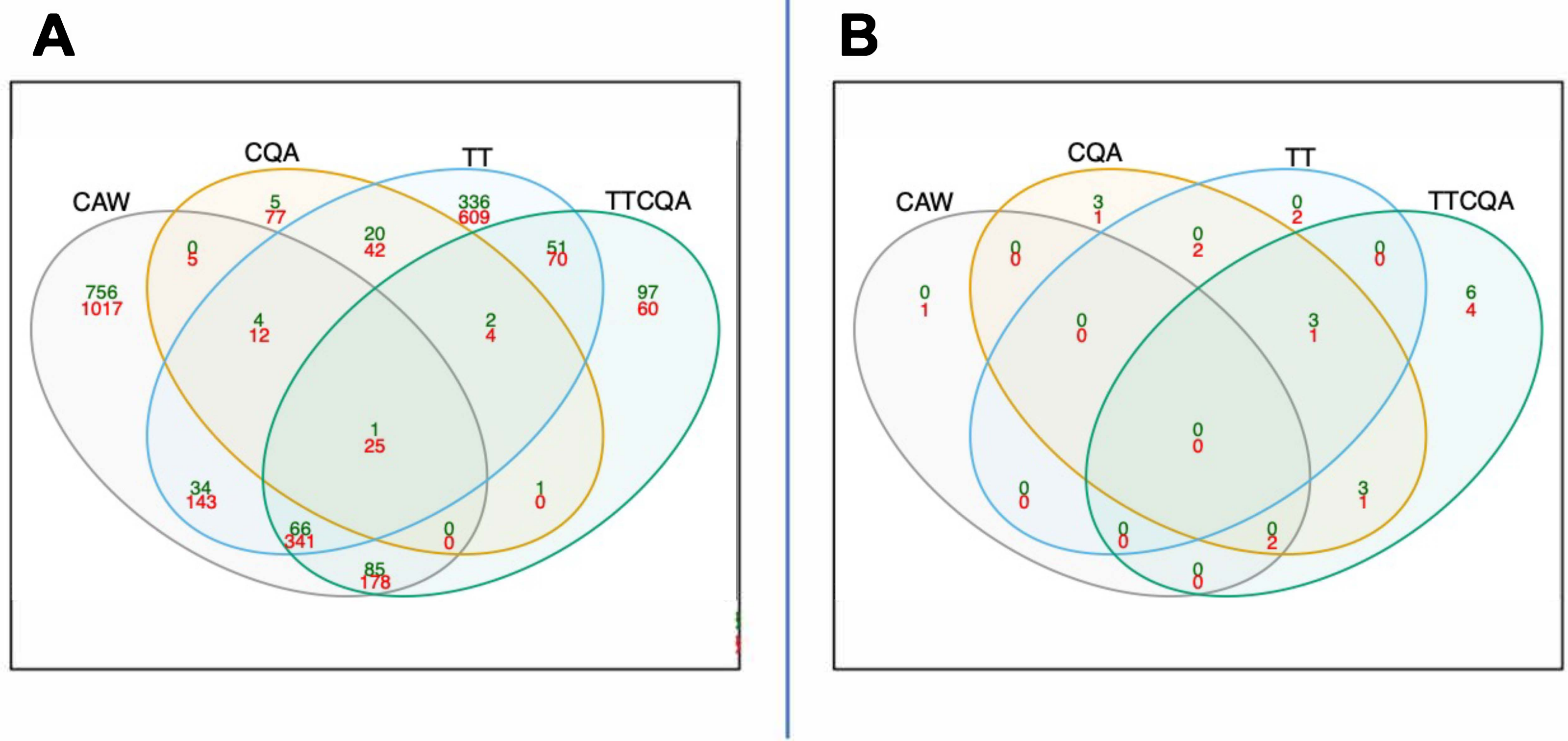

Figure S1. Molecular effects of the four treatments. Overlap of differential analyses for gene expression **(A)** and metabolite abundance **(B)** for each of the four treatments, relative to control vehicle. The number of upregulated genes/metabolites are shown in green and downregulated genes/metabolites are shown in red.
