# Supplementary material for: Multi-Omics Analysis in Mouse Primary Cortical Neurons Reveals Complex Positive and Negative Biological Interactions Between Constituent Compounds of Centella asiatica
Source: Pharmaceuticals (Basel). 2024 Dec 27;18(1):19. doi: 10.3390/ph18010019 (PMC11768890; doi:10.3390/ph18010019)
Supplement: Supplementary file 1 [file pharmaceuticals-18-00019-s001.zip › Figure S7.pdf]

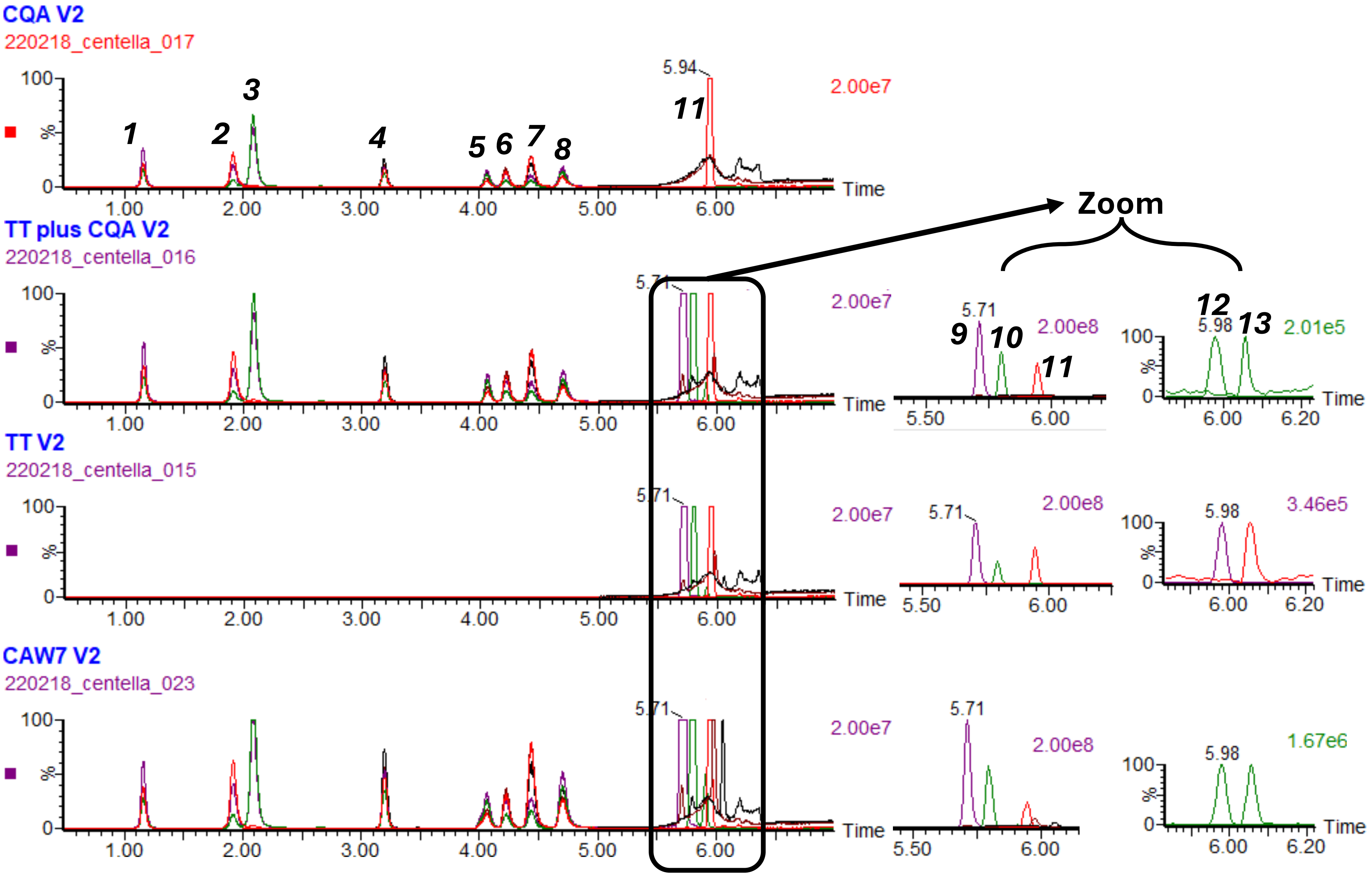

Figure S7. LC-MRM-MS of stock solutions used for preparation of media. Treatment groups are indicated on graphs as CQA V2: caffeoylquinic acids only, TT plus CQA V2: triterpenes and caffeoylquinic acids, TT V2: triterpenes only, and CAW7 V2: A water extract of *centella asiatica*.

1. 5-caffeoylquinic acid
2. 4-caffeoylquinic acid
3. 3-caffeoylquinic acid
4. 1,3-Dicaffeoylquinic acid
5. 3,4-Dicaffeoylquinic acid
6. 3,5-Dicaffeoylquinic acid
7.1,5-Dicaffeoylquinic acid
8. 4,5-Dicaffeoylquinic acid
9. Madecassoside
10. Asiaticoside
11. Digoxin-d3
12. Madecassic Acid
13. Asiatic Acid
