# Supplementary material for: Multi-Omics Analysis in Mouse Primary Cortical Neurons Reveals Complex Positive and Negative Biological Interactions Between Constituent Compounds of Centella asiatica
Source: Pharmaceuticals (Basel). 2024 Dec 27;18(1):19. doi: 10.3390/ph18010019 (PMC11768890; doi:10.3390/ph18010019)
Supplement: Supplementary file 1 [file pharmaceuticals-18-00019-s001.zip › Figure S8.pdf]

Figure S8: UPLC-MS analysis of CAW extract and compounds. Upper figure: UPLC-qTOF data dependent acquisition (DDA)-MS showing numerous peaks in addition to the triterpenes and CQAs. Lower figure: UPLC-MRM-MS of TT and CQA standards.

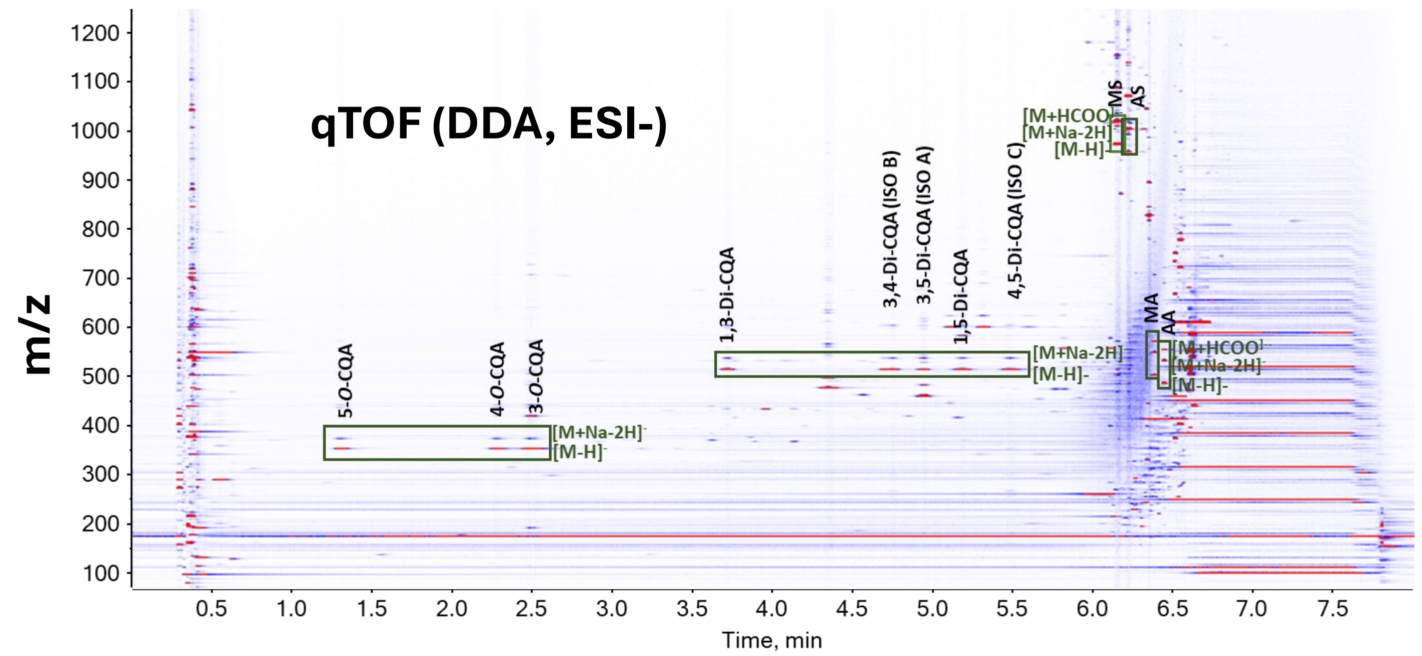

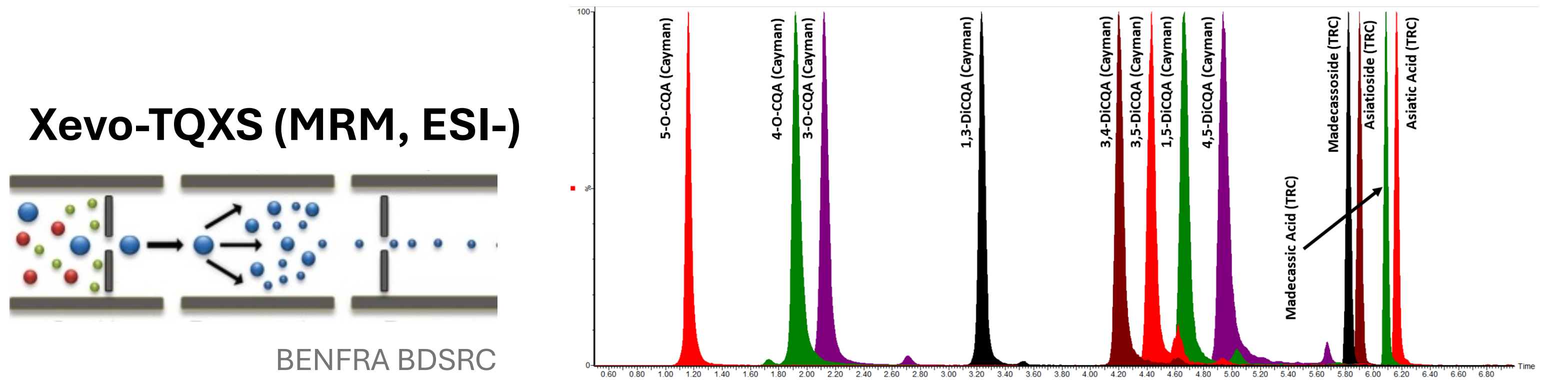

Current UPLC Method:
Inertsil **Phe-3**, 2 micron, 100 x 2.1 mm
A: Water + 0.1% FA; B: MeOH + 0.1% FA
Flow 0.8 mL/min, T= 55 °C
