# Supplementary material for: Multi-Omics Analysis in Mouse Primary Cortical Neurons Reveals Complex Positive and Negative Biological Interactions Between Constituent Compounds of Centella asiatica
Source: Pharmaceuticals (Basel). 2024 Dec 27;18(1):19. doi: 10.3390/ph18010019 (PMC11768890; doi:10.3390/ph18010019)
Supplement: Supplementary file 1 [file pharmaceuticals-18-00019-s001.zip › Table S1.pdf]

Supplemental Table S1. Concentration (µM) of phytochemical compounds in media cultured with neurons quantified using LC-MRM-MS.

The first four lines show expected starting concentrations (at time 0) of phytochemical marker compounds calculated from LC-MRM-MS analysis of concentrated stock solutions which were diluted 1/1000 into cell culture media. Phytochemical markers in 48h cultured media (CM) were measured in three biological replicates for each additive. All concentrations are reported as mean of triplicate injections of each sample. Abbreviations: <LOD = less than limit of detection defined as 3 x standard deviation of the noise; <LOQ = less than limit of quantification defined as 10 x standard deviation of the noise; 5-CQA = 5-caffeoylquinic acid: 4-CQA = 4-caffeoylquinic acid; 3-CQA = 3-caffeoylquinic acid; 1,3-DiCQA =1,3-dicaffeoylquinic acid; 3,4-DiCQA = 3,4-dicaffeoylquinic acid; 3,5-DiCQA =3,5-dicaffeoylquinic acid; 1,5-DiCQA =1,5-dicaffeoylquinic acid; 4,5-DiCQA = 4,5-dicaffeoylquinic acid; MS = madecassoside; AS = asiaticoside; MA = madecassic acid; AA = asiatic acid.

Sample ID	5-CQA	4-CQA	3-CQA	1,3-DiCQA	3,4-DiCQA	3,5-DiCQA	1,5-DiCQA	4,5-DiCQA	MS	AS	MA	AA
CAW Expected T0	0.48827477	0.48150102	1.18202009	0.29296156	0.26023816	0.17852648	0.39151902	0.18530351	1.3848552	0.63766786	0.06578165	<LOQ
TT Expected T0	<LOD	<LOD	<LOD	<LOD	<LOD	<LOD	<LOD	<LOD	1.31265895	0.41100175	0.02991876	<LOQ
CQA Expected T0	0.60173515	0.55657679	1.40498949	0.25229935	0.25694646	0.21221803	0.3808694	0.16942589	<LOD	<LOD	<LOD	<LOD
TTCQA Expected T0	0.61725834	0.57322894	1.46567104	0.27185594	0.28056927	0.21996321	0.41262465	0.163617	1.46987037	0.72535241	0.00990688	<LOD
CAW7_CM_1	0.12621762	0.08495417	0.08461548	0.02784393	0.03440798	0.01396069	0.01022364	0.02534611	1.91305685	0.82027275	0.13253418	0.06081441
CAW7_CM_2	0.04882748	0.02540158	0.0344897	0.0041824	0.01506438	0.00714493	0.00762901	0.00944912	1.98267905	0.85418926	0.13003765	0.06331082
CAW7_CM_3	0.04806543	0.02503467	0.03130039	0.0062736	0.01504502	0.00735792	0.0069513	0.01022364	1.97992042	0.86525148	0.1301169	0.06214446
CQA_CM_1	0.62431433	0.51793817	0.47966646	0.1920031	0.1488237	0.06085778	0.03514377	0.08513893	<LOQ	<LOQ	<LOQ	<LOD
CQA_CM_2	0.75665655	0.63755138	0.57480948	0.30239133	0.17112983	0.07731629	0.04308258	0.09210959	<LOD	<LOD	<LOQ	<LOD
CQA_CM_3	0.66284006	0.54833539	0.51949049	0.19465582	0.16830284	0.06643431	0.0400426	0.09667925	<LOQ	<LOQ	<LOQ	<LOD
TTCQA_CM_1	0.56055637	0.47218711	0.43656845	0.19004744	0.13248136	0.05574596	0.0341369	0.07491529	1.66867668	0.7483318	0.04214385	0.00620012
TTCQA_CM_2	0.85885557	0.73396448	0.65321568	0.38371575	0.19740536	0.08883725	0.04430245	0.10636073	1.91836902	0.86620027	0.04965326	0.00918764
TTCQA_CM_3	0.43233485	0.35480359	0.34551791	0.12247071	0.1112402	0.04407009	0.03086456	0.06428502	1.64627943	0.75015639	0.05496334	0.00816452
TT_CM_1	<LOD	<LOD	<LOD	<LOD	<LOD	<LOD	0.00456966	<LOD	1.66223644	0.80492535	0.04176739	0.0153673
TT_CM_2	<LOD	<LOD	<LOD	<LOD	<LOD	<LOD	0.00431794	<LOD	1.71835261	1.07096088	0.04148999	0.01943933
TT_CM_3	<LOD	<LOD	<LOD	<LOD	<LOD	<LOD	0.00441475	<LOD	1.63098901	0.76546209	0.03869626	0.01559239
MeOH_CM_1	<LOD	<LOD	<LOD	<LOD	<LOD	<LOD	0.00422112	<LOD	<LOD	<LOD	<LOQ	<LOD
MeOH_CM_2	<LOD	<LOD	<LOD	<LOD	<LOD	<LOD	0.00443412	<LOD	<LOD	<LOD	<LOQ	<LOD
MeOH_CM_3	<LOD	<LOD	<LOD	<LOD	<LOD	<LOD	0.00445348	<LOD	<LOD	<LOD	<LOQ	<LOD
